# iTRAQ plasma proteomics analysis for candidate biomarkers of type 2 incipient diabetic nephropathy

**DOI:** 10.1186/s12014-019-9253-1

**Published:** 2019-07-31

**Authors:** Hongmei Lu, Shaodong Deng, Minghui Zheng, Kunhua Hu

**Affiliations:** 1 The Second Clinical Medical College, Guangdong Medical University, Dongguan, 523808 China; 2 Department of Clinical Laboratory, Sun Yat-sen Memorial Hospital of Sun Yat-sen University, Guangzhou, 510120 China; 3 Proteomics Center, Zhongshan School of Medicine, Sun Yat-sen University, Guangzhou, 510080 China

**Keywords:** Incipient diabetic nephropathy, Biomarker, iTRAQ

## Abstract

**Background:**

Diabetic nephropathy is the most frequent cause of end-stage renal disease worldwide. Identification of biomarkers for diabetic nephropathy for early diagnosis may be the key to avoiding damage from this condition.

**Methods:**

Proteomic iTRAQ technology was first used to identify differentially expressed plasma proteins in type 2 incipient diabetic nephropathy (IDN) using a Q-Exactive mass spectrometer.

**Results:**

Compared with controls, 57 proteins (32 upregulated and 25 downregulated proteins) were identified. Furthermore, the gelsolin, collectin-11, PTPRJ, and AKAP-7 proteins were confirmed by Western blots as candidate biomarkers for type 2 IDN through ROC analysis.

**Conclusions:**

These findings offer a theoretical basis for the early treatment of diabetic nephropathy.

## Background

The total number of type 2 diabetes patients is expected to increase to 366 million in 2030 [1]. Diabetic nephropathy (DN) occurs in 30% to 40% of type 2 diabetic patients according to the classification of the American Diabetes Association [2] and accounts for approximately half of all new cases of end-stage renal disease that require dialysis or transplantation treatment [3, 4]. DN is divided into three stages: incipient nephropathy (microalbuminuria), clinical diabetic nephropathy (macroalbuminuria) and end-stage kidney damage. Microalbuminuria is used for the early detection of diabetic renal damage, and intervention at the incipient nephropathy stage can effectively prevent the progression to end-stage kidney damage [5]. However, some structural destruction has already occurred when microalbuminuria is observed [6]. Nephropathy also develops in other diseases, such as cardiovascular disease, inflammation and hypertension [7]. Meanwhile, many patients who already have advanced renal histopathological changes show normoalbuminuria [8]. Therefore, more accurate biomarkers for IDN are required.

Urinary proteomic technology has been applied to study biomarkers for type 2 DN. For example, Jin et al. found that alpha-1-antitrypsin, alpha-1-acid glycoprotein 1 and prostate stem cell antigen were biomarker candidates for DN using urinary analysis [7]. Guo et al. found that DN was associated with alpha-1-antitrypsin and ceruloplasmin through urinary glycoprotein analysis [5]. To date, more than 200 differentially expressed proteins have been reported in DN urine using various proteomic methods. Efforts to distinguish DN based on urine biomarkers need to be further validated and may suffer from sampling variability and signal suppression by albumin. In addition to urine, plasma has been widely applied in studying biomarkers for various diseases and may have a larger dynamic range for the proteins of interest by removal of peak proteins [9]. Therefore, plasma is also the preferred media for early diagnosis of DN because of its ease of collection [10]. In this study, we first identified differentially expressed proteins in the plasma of type 2 IDN patients using the iTRAQ technique.

## Methods

### Collection of plasma samples

Plasma samples from 164 people were collected according to the diagnostic criteria for type 2 incipient diabetic nephropathy (Guidelines for the Prevention and Treatment of Diabetes in China Refer to International Mogensen Staging Standards) as follows: (1) Type 2 diabetes mellitus (compliance with the diagnostic criteria of diabetes mellitus in the 1999 WHO expert consultation report) a. diabetic symptoms and plasma glucose levels at any time ≥ 11.1 mmol/L (200 mg/dL), FPG ≥ 7.0 mmol/L (126 mg/dL), or OGTT test, 2 hPG ≥ 11.1 mmol/L (200 mg/dL). (2) Two or more continuous urinary albumin excretion rates within 6 months were 30–300 mg/24 h (Table 1). Other possible causes of nephropathy, including ketoacidosis, urinary tract infection, hypertension, obesity and heart failure, were excluded. All samples were provided by the First Affiliated Hospital of Guangzhou University of Chinese Medicine and Sun Yat-sen Memorial Hospital of Sun Yat-sen University. Written informed consent was obtained from each participant before the commencement of this study, and The Human Research Ethics Committee from Guangzhou University of Chinese Medicine and Sun Yat-sen University approved all aspects.

**Table 1:** Clinical characteristic of normoalbuminuria and microalbuminuria in type 2 diabetic patients

Characteristics	Microalbuminuria (IDN)	Normoalbuminuria (control)	p value
n (male/female)	82 (42/40)	82 (42/40)	–
Age (years)	59 ± 10	61 ± 8	0.53
DN duration (years)	5.2 ± 4.3	4.5 ± 3.7	0.43
BMI (kg/m^2^)	24.12 ± 6.23	23.52 ± 5.71	0.55
HbA1c (%)	9.02 ± 5.31	8.01 ± 6.82	0.48
Creatinine (μ mol/L)	78.20 ± 11.01	80.43 ± 10.32	0.42
CCr (mL/min)	85.30 ± 12.22	87.30 ± 10.75	0.56
UAE (mg/24 h)	210.64 (128.32–261.26)	11.37 (6.54–25.31)	<0.0001
ACR (mg/g)	81.31 (43.62–227.51)	16.40 (11.27–20.22)	<0.001

*BMI* body mass index, *HbA1c* haemoglobin A1c, *CCr* creatinine clearance rate; *UAE* 24-h urinary protein quantification, *ACR* the ration of Urinary albumin excretion rate/urinary microalbumin creatinine

p value < 0.05 was considered to indicate statistical significance

### iTRAQ labeling

Aliquots of plasma samples from ten randomly selected individuals were mixed into four pools. Groups N1 and N2 (N = controls) and groups IDN1 and IDN2 (IDN patients) were formed. Then, they were processed using the ProteoPrep Blue Albumin Depletion Kit (Sigma, St. Louis, MO, USA), and protein samples (100 μg) were digested into peptides with trypsin (Promega, USA). Next, iTRAQ labeling was performed according to the manufacturer’s instructions (Applied Biosystems Sciex, #4381664): N1-iTRAQ 115 reagent, N2-iTRAQ 116 reagent, IDN1-iTRAQ 117 reagent, IDN2-iTRAQ 118 reagent. Finally, the labeled samples were mixed together and vacuum dried.

### Mass spectrometric analysis

Mass spectrometric analysis was carried out as previously reported [11]. The iTRAQ-tagged peptides were reconstituted and loaded onto Gemini NX-C18 columns using a Dionex UltiMate 3000 HPLC system. Then, nano LC–MS/MS was carried out by Q Exactive, and MS data were acquired using a data-dependent top 20 method, dynamically choosing the most abundant precursor ions for HCD fragmentation analysis for 60 min. Protein Pilot 5.0 (AB Sciex, USA) was used for protein identification and quantification analysis. Database searching parameters were as follows: sample type: iTRAQ 8plex (peptide labeled), Cys alkylation: MMTS, digestion: trypsin, FDR < 1%, T-test was used to identify significant (p < 0.05) differences in means between IDN and controls with an average ratio-fold change ≥ 1.5 or ≤ 0.66. A minimum of two peptides matches in common was confidently considered differential expression of proteins.

### Bioinformatics analysis

GO annotation was carried out to understand the biological function of differentially expressed proteins, which includes three main modules as follows: biological process, cellular component and molecular function (http://www.geneontology.org). STRING analysis (http://www.string-db.org) was used for protein–protein interaction networks, including direct (physical) and indirect (functional) associations.

### Western blot and ROC analysis

Proteins (80 µg) were separated using SDS-PAGE electrophoresis and were transferred onto PVDF membranes. Primary antibodies against gelsolin (Abcam, #109014), transthyretin (Abcam, #92469), pregnancy zone protein (Proteintech, #21742), A-kinase anchor protein 7 (Proteintech, #12591), PTPRJ (Proteintech, #55123), and collectin-11 (Proteintech, #15269) were incubated overnight, followed by the appropriate horseradish peroxidase-conjugated secondary antibodies. All blots were visualized using ECL. Quantification was performed using ImageJ software. The statistical analyses were performed using GraphPad Prism version 6.01 (https://www.graphpad.com). Student’s t-test was applied for comparisons of quantitative data, and the ROC analysis with SPSS statistics 20.0 software was performed to evaluate the sensitivity and specificity of each protein.

## Results

### Clinical data

Table 1 shows the clinical data regarding normoalbuminuria (control group) and microalbuminuria (IDN group) in type 2 diabetic patients. Compared to the control group, patient age, duration of diabetes mellitus, BMI, HbA1c, creatinine, and creatinine clearance rate had no significant differences in the IDN group (p value > 0.05). However, UAE and ACR showed significant differences between the two groups (p value < 0.05).

### Basic data of mass spectrometry

**Fig. 1:**
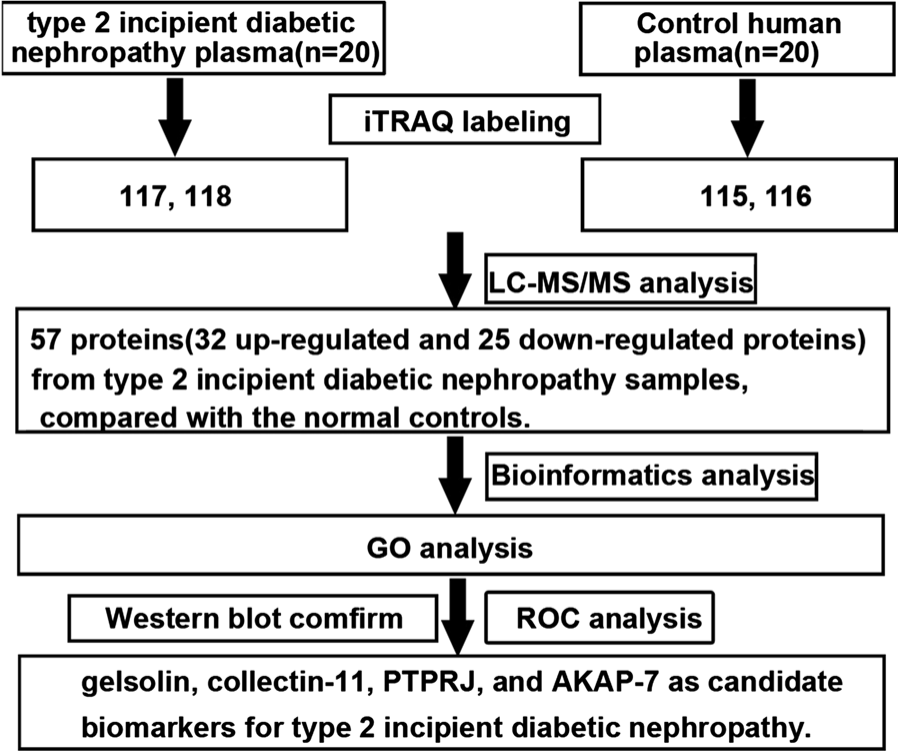
The experimental plan of the present study. iTRAQ was applied to identify the differentially expressed proteins in type 2 incipient diabetic nephropathy. Gelsolin, collectin-11, PTPRJ and AKAP-7 proteins were candidate biomarkers for type 2 incipient diabetic nephropathy

**Fig. 2:**
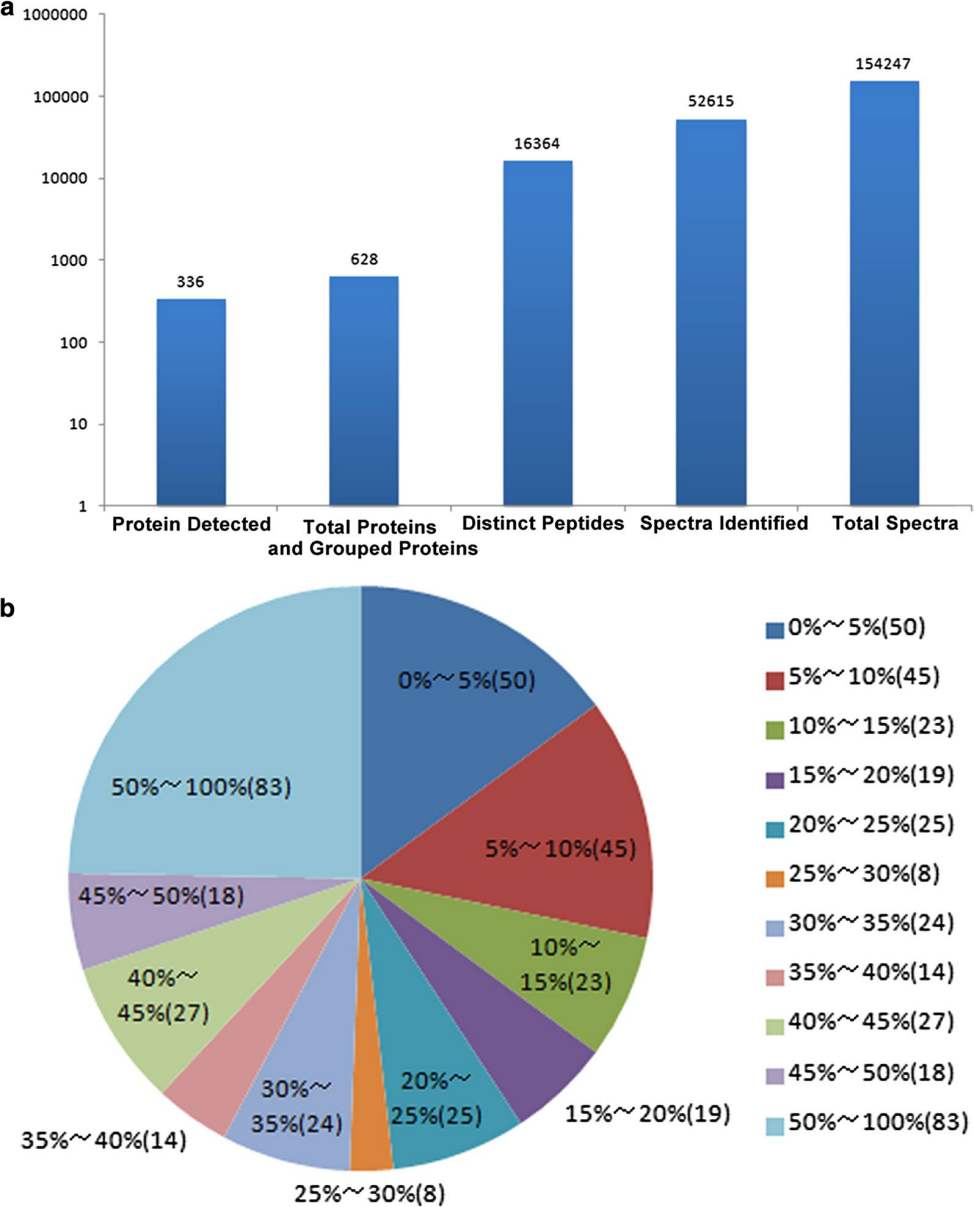
Basic data of the type 2 incipient diabetic nephropathy proteome. **a** Total spectra, spectra identified, distinct peptides, proteins before grouping, and proteins detected were acquired from iTRAQ analysis. **b** The identified proteins were classified into pie charts according to the protein’s sequence coverage

The experimental plan to identify potential biomarkers for type 2 IDN is shown (Fig. 1). A total of 154,247 spectra and 336 proteins were acquired using ProteinPilot™ software 5.0.1 (Fig. 2a). Protein sequence coverage of 50–100% and 30–50% variation accounted for 83 and 83 proteins, respectively (Fig. 2b). Then, 32 proteins increased by more than 1.5-fold, and 25 proteins decreased to less than 0.66-fold in type 2 IDN patients compared to controls (p-values ≤ 0.05 and FDR < 1%) (Table 1).

### GO annotation

**Fig. 3:**
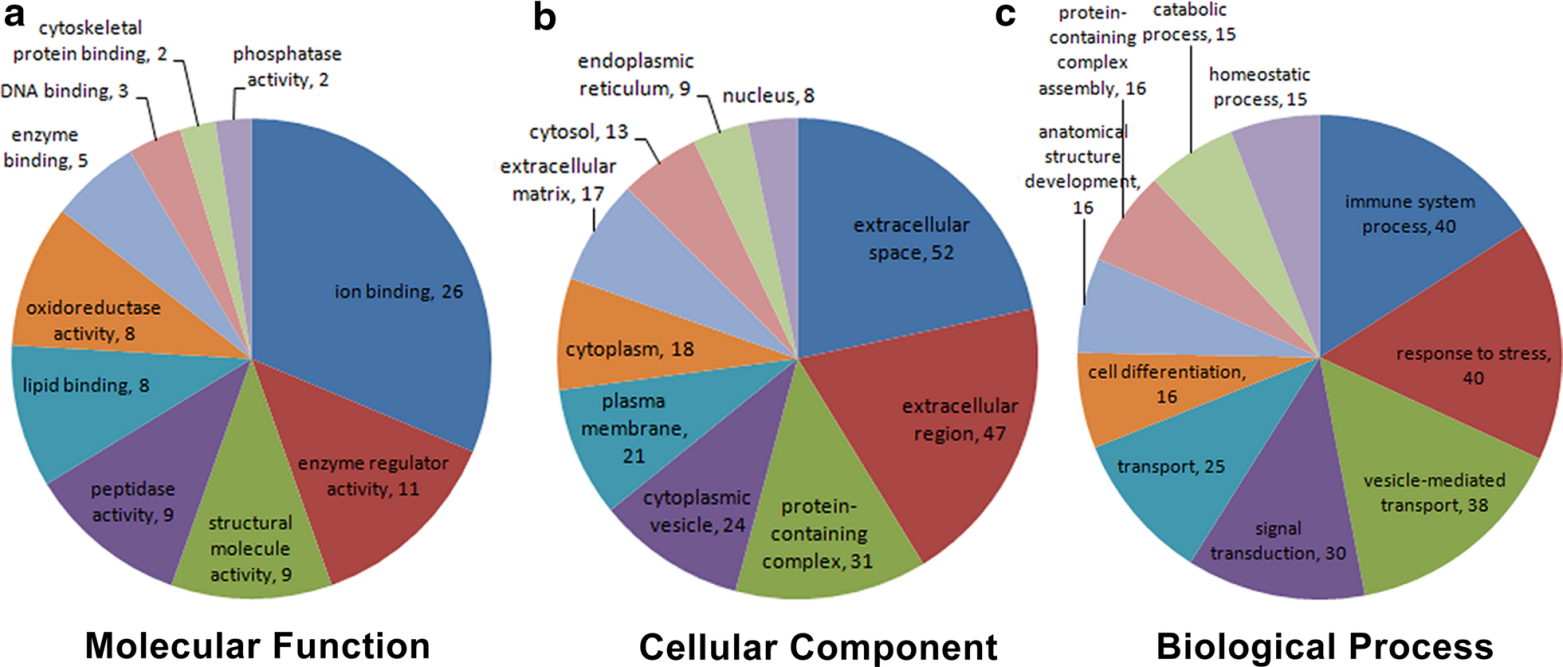
GO annotation of differential expression of proteins in type 2 incipient diabetic nephropathy. **a** Molecular function. **b** Cellular component. **c** Biological process

Differentially expressed proteins were classified into three categories using GO annotation, including molecular functions: response to ion binding (26%), enzyme regulator activity (11%) and structural molecule activity (9%) (Fig. 3a); cellular components: extracellular space (52%), extracellular region (47%), protein-containing complex (31%) and cytoplasmic vesicle (24%) (Fig. 3b); and biological processes: immune system process (40%), stress (40%), vesicle-mediated transport (38%) and signal transduction (30%) (Fig. 3c).

### STRING analysis

**Fig. 4:**
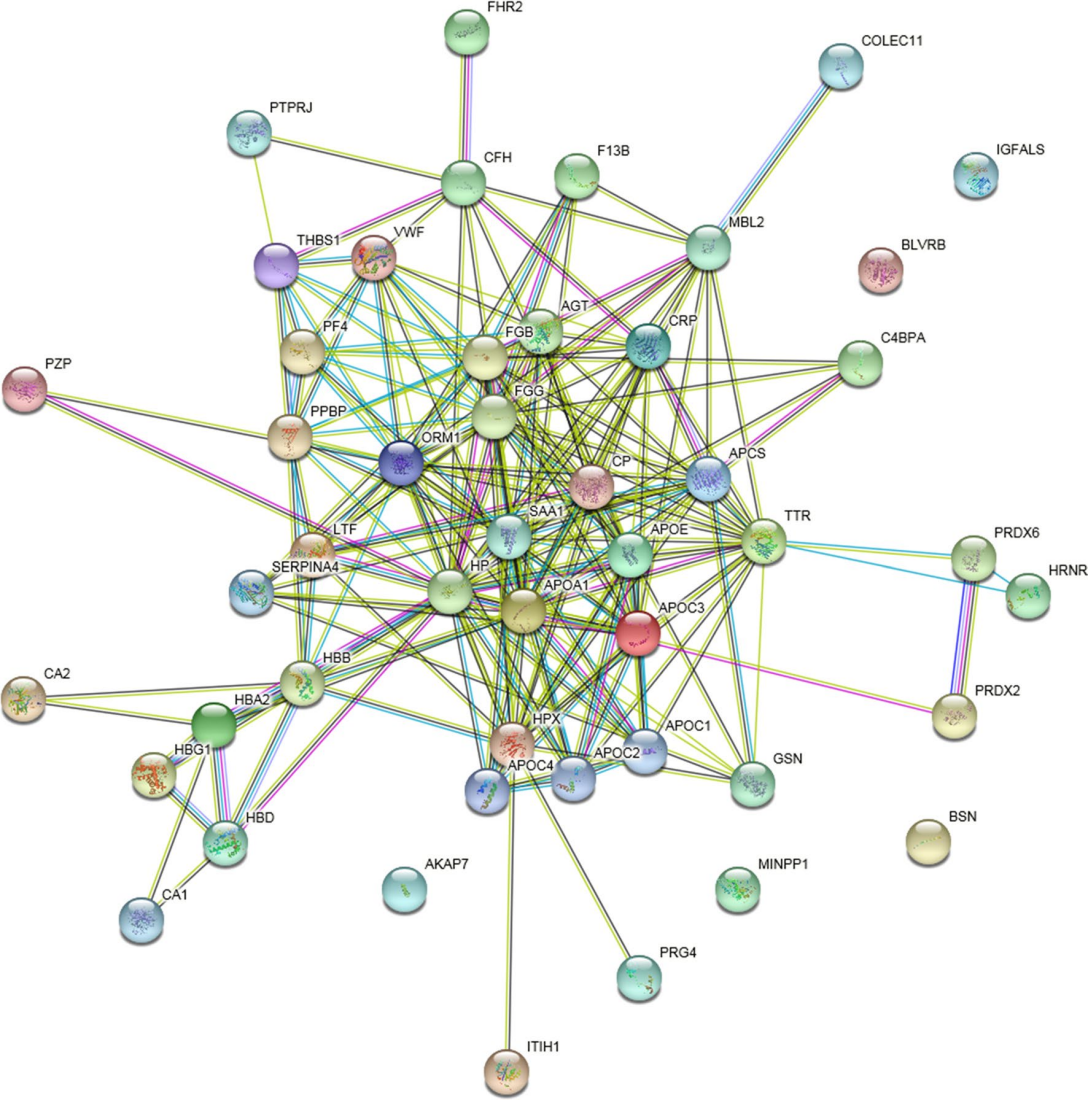
Interaction network analysis. Nodes are proteins, lines represent functional associations between proteins and different line colors represent the types of evidence for the predicted functional association

Biological functions can be regulated as a complex network with protein–protein interactions. To better understand the pathogenic mechanisms in IDN, STRING was constructed for the identified variable proteins (Fig. 4), and many proteins were at the core of the “traffic link”, such as gelsolin, collectin-11, PTPRJ, and pregnancy zone protein, which suggested that they may play a key role in the development of IDN.

### Validation with Western blot analysis

**Fig. 5:**
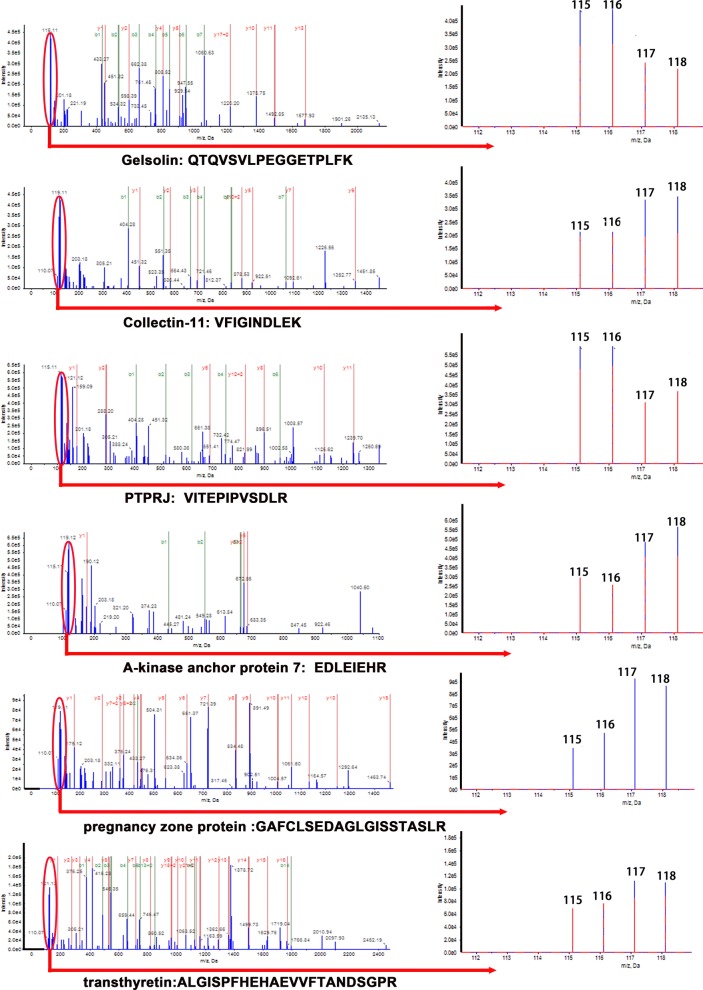
MS/MS spectrum for iTRAQ quantification. A representative MS/MS spectrum indicated important peptide segments for gelsolin, collectin-11, PTPRJ, AKAP-7, pregnancy zone protein and transthyretin. iTRAQ tags showed the relative expression of the six proteins individually in the plasma of type 2 incipient diabetic nephropathy patients compared to the control

**Fig. 6:**
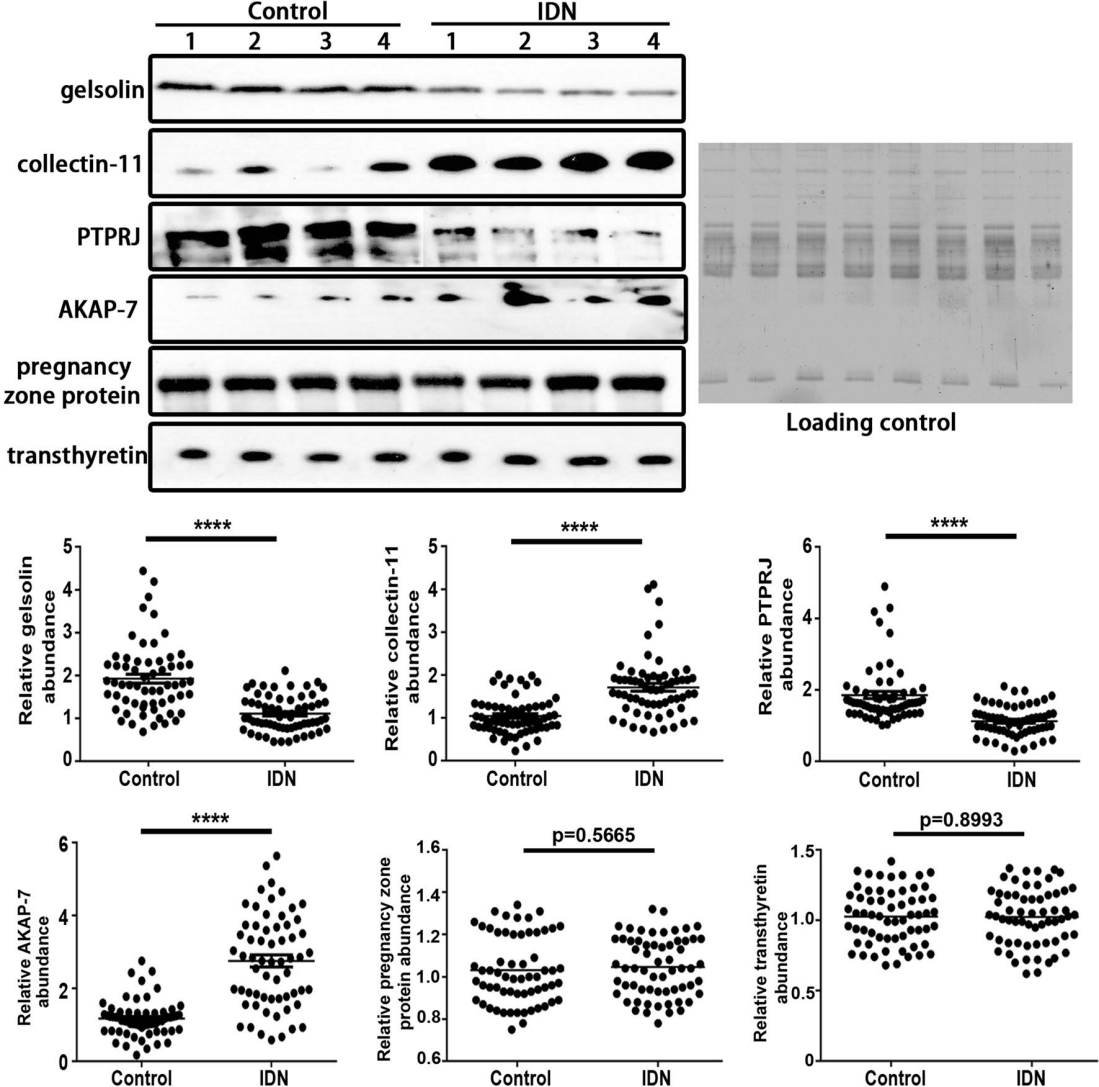
Western blot confirmation of six selected proteins. Representative WB images for six proteins, including gelsolin, collectin-11, PTPRJ and AKAP-7, pregnancy zone protein and transthyretin. Data are expressed as the mean using GraphPad Prism. ****p < 0.0001. Controls (n = 62), IDN patients (n = 62)

To identify new potential markers, the function of differentially expressed proteins was analyzed through PubMed literature and String analysis. Finally, six proteins (gelsolin, collectin-11, PTPRJ, AKAP-7, pregnancy zone protein, and transthyretin) were further verified using Western blots and iTRAQ quantification in the MS/MS spectrogram in Fig. 5. The WB showed that the ratio of collectin-11 and AKAP-7 increased to 1.71 (p < 0.0001) and 2.76 (p < 0.0001). Furthermore, the ratio of gelsolin and PTPRJ decreased to 0.58 (p < 0.0001) and 0.61 (p < 0.0001), respectively, in type 2 IDN. Western blot results showed the same trend of change with iTRAQ methods. However, pregnancy zone protein and transthyretin showed no significant differences between type 2 IDN patients and controls (Fig. 6).

### ROC diagnosis

**Fig. 7:**
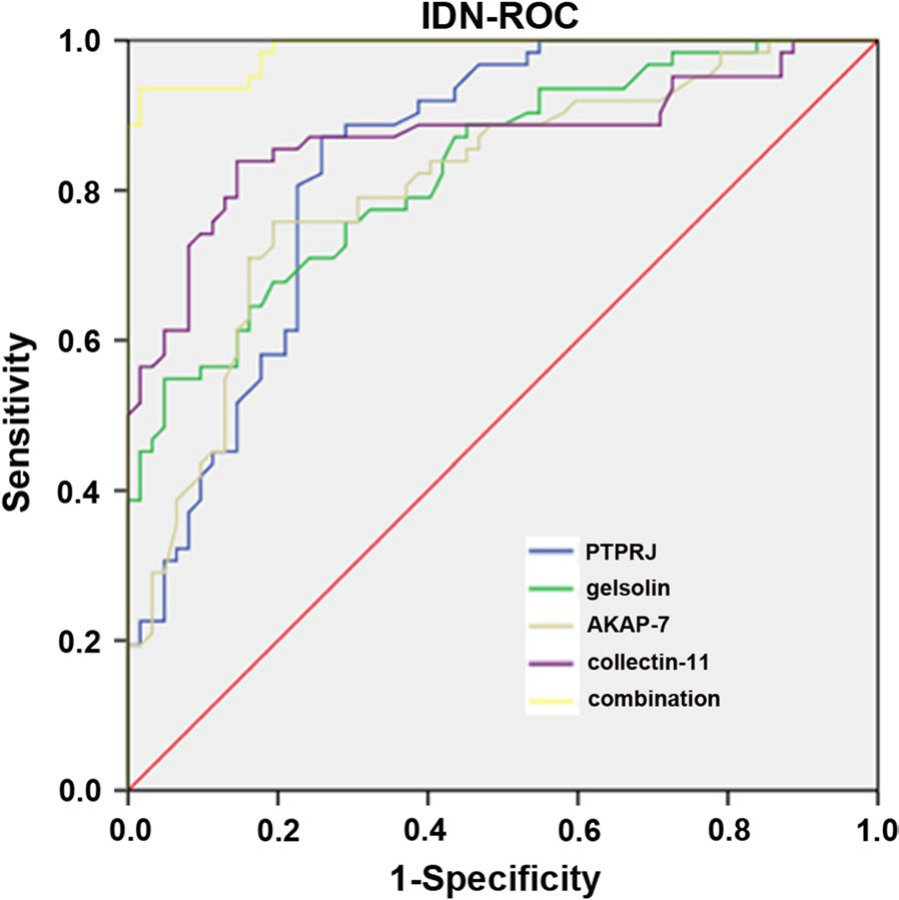
ROC curve analysis for the specificity and sensitivity of gelsolin alone, collectin-11 alone, PTPRJ alone and AKAP-7 alone, and their combination in the diagnosis of type 2 incipient diabetic nephropathy

ROC analysis was performed to evaluate the specificity and sensitivity of each confirmed protein. The accuracy of the area under the ROC curve was assessed: 0.9–1 = excellent, 0.8–0.9 = good, 0.7–0.8 = fair, and < 0.7 = not useful. ROC analysis identified gelsolin (AUC: 0.828; 95% CI 0.758–0.898), collectin-11 (AUC: 0.807; 95% CI 0.730–0.884), PTPRJ (AUC: 0.839; 95% CI 0.769–0.910), and AKAP-7 (AUC: 0.872; 95% CI 0.805–0.939). The combination analysis of these four proteins (AUC: 0.988; 95% CI 0.975–1.000) had a significant predictive value for type 2 IDN (Fig. 7).

## Discussion

DN, which manifests as glomerular basement membrane thickening, matrix deposition and proteinuria, is one of the most serious and common chronic complications of diabetes mellitus.

Its pathogenesis is extremely complex and is caused by hyperglycemia under a certain genetic background, involving the polyol pathway (aldose reductase) [12], PKC activation pathway [13], oxidative stress (ROS) [14], immune inflammation [15] and so on.

Haptoglobin (Hp) is a circulating glycoprotein that is mainly synthesized in the liver [16], which reduces the oxidative ability of heme iron by scavenging free hemoglobin [17]. Hp has been identified as a candidate biomarker for several human diseases, such as acute allograft rejection [18], chronic renal insufficiency [19] and diabetic nephropathy [20]. Urinary Hp is associated with diabetic retinopathy for predicting kidney damage in patients with Type 2 diabetes mellitus (T2D) [19]. Hp and α-1-microglobulin distinguished diabetic nephropathy in Taiwanese patients through urinary proteomics [21]. Urinary Hp can also improve the prediction ability of albuminuria for renal progression in Asians with T2D [22]. Bhensdadia et al. reported that urinary Hp and albuminuria together as prognostic biomarkers appeared to provide better diagnostic accuracy for progressive DN, and the urine Hp to creatinine ratio was useful for predicting T2D nephropathy prior to macroalbuminuria or changes in the glomerular filtration rate [23]. In our study, we found that Hp was also sharply upregulated in IDN plasma (4.5-fold vs. control), consistent with the change in urine Hp, as plasma Hp can leak into urine when the glomerular permeability is reduced to a certain level, which indicated that plasma Hp could be a good indicator for IDN.

Plasma gelsolin (pGSN) is mainly synthesized and secreted by skeletal, cardiac, and smooth muscles [24]. pGSN is a multifunctional protective protein, and pGSN may reduce the release of actin into systemic circulation in response to injury or necrosis, resulting in adverse pathophysiologic consequences [25]. pGSN can also diminish the inflammatory response by preventing Toll-like receptor activation [26] and lower oxidative stress due to limited myeloperoxidase activity [27]. pGSN levels are reduced in many human diseases, including trauma, neurodegenerative diseases, cancers, chronic kidney diseases, and diabetes. Depletion of pGSN led to the release of actin for tissue damage and cell death, and recombinant gelsolin might serve as a treatment for diabetes [28]. pGSN is strongly negatively correlated with IgA nephropathy, and pGSN is also defined as a biomarker for detecting early stage diabetic retinopathy through the recruitment and activation of inflammatory factors [29]. pGSN also appeared to be a promising diagnostic marker for diabetes mellitus using urine proteomics [30]. Decreased gelsolin may cause kidney damage by promoting PKC activation [31], which involves the production of ROS [32] and inflammation [33] in DN. In short, a decline in pGSN might be an indicator of critical conditions, although it is not a specific biomarker for a particular disease; meanwhile, pGSN as a combined biomarker, together with other proteins, is able to augment the sensitivity and specificity of GSN-based biomarkers [34]. In our study, pGSN was downregulated in the IDN group, and ROC analysis (AUC: 0.828; 95% CI 0.758–0.898) indicated that pGSN could be a good indicator in diabetes of diabetic nephropathy complications.

The innate immune system plays an important role in host defense. Complement activation, including the lectin pathway, is an important constituent of the innate immune system. Collagen-like lectins (collectins) are a family of collagenous Ca^2+^-dependent lectins that are highly conserved among species [35]. There are three classical collectins in humans: serum mannose-binding lectin (MBL), surfactant protein A, and surfactant protein D. Collectin liver 1 (collectin-10), collectin kidney 1 (collectin-11) and collectin placenta 1 (collectin-12) are the most recently discovered collectins and play important roles in host defense by recognizing a variety of microorganisms and interacting with complement components [36]. MBL, which was identified as the first molecule of the lectin pathway, has a double role in humans: sometimes its presence is associated with pathological deterioration, while in other cases it is an important part of the body defense system [37]. MBL is associated with many human diseases: low MBL can identify neonates with inflammatory response syndrome when developing sepsis [38]; MBL can serve as an inflammatory marker in predicting the prognosis of patients with community-acquired pneumonia [39]; elevated MBL can be used as an independent marker for diabetic retinopathy [40]; and low MBL is predictive for coronary artery disease and myocardial infarction [41]. Collectin-11, which is mainly expressed in the adrenals, kidneys and livers, is a useful marker for schistosomiasis investigations [42]. Low collectin-11 is associated with an increased risk of infection or colonization [43]. Deficiency of collectin-11 was shown to cause the rare autosomal recessive syndrome known as 3MC (Mingarelli, Malpuech, Michels and Carnevale Syndrome) [44]. Elevated collectin-11 may serve as a biomarker for disseminated intravascular coagulation and respiratory disorders [45]. Collectin-11 also activated the innate defense system in urinary tract and kidney diseases, and collectin-11-deficient mice were protected against loss of renal function due to reduced complement deposition [35]. In the present study, collectin-11 was elevated in IDN and may promote lectin pathway activation to impair renal function. Meanwhile, ROC analysis (AUC: 0.807; 95% CI 0.730–0.884) showed that collectin-11 could be a better indicator of progression from diabetes to diabetic nephropathy complications.

Protein-Tyrosine Phosphatase Receptor Type J (PTPRJ) is a tumor suppressor that negatively regulates processes such as angiogenesis, cell proliferation and migration and is widely expressed in many cell types, including epithelial and endothelial cells [46]. PTPRJ is a candidate colorectal cancer susceptibility gene, and loss of PTPRJ is an early event in colorectal tumorigenesis and thyroid [47]. PTPRJ is a negative regulator of FLT3 signaling that is linked to ROS formation and DNA damage, and deletion of PTPRJ promoted myeloproliferative disease in FLT3-internal tandem duplication acute myeloid leukemia [48]. Overexpression of PTPRJ is associated with inhibition of cell proliferation and migration in many tumor cells [49, 50]. PTPRJ is also the key endogenous regulator of inflammation and a therapeutic target for inflammatory diseases [51]. Missense polymorphisms of the PTPRJ gene affect susceptibility to a variety of human cancers, including lung, head and neck, colorectal, and esophageal cancers [52]. Furthermore, PTPRJ is critical for clear renal cell carcinoma development [53]. PTPRJ was downregulated in IDN, which accelerates autoimmunity through ROS and inflammation. ROC analysis (AUC: 0.839; 95% CI 0.769–0.910) indicated that PTPRJ could be a good candidate marker.

A kinase anchor proteins (AKAPs) are functionally related scaffolding proteins that target protein kinase A (PKA) and other enzymes in signal transduction [54]. Disturbance or mutation of AKAPs results in unregulated signaling associated with oncogenesis, cancer maintenance, and metastasis [55]. Downregulation of AKAP12 may act as a candidate tumor biomarker of several malignancies, such as prostate cancer, breast cancer, gastric cancer and hepatocellular carcinoma [56, 57]. Meanwhile, AKAP3 and AKAP13 may be potential biomarkers for the diagnosis and prognosis of hepatocellular carcinoma [58, 59]. AKAP4 is a cancer testes antigen that can be detected in cervical, ovarian, breast and prostate cancers [60, 61]. Gravin promoter methylation is a potential biomarker for cancer progression [62]. AKAP-7 is a low molecular weight AKAP whose functions are also involved in regulating PKA. Cardiac AKAP-7, which is located in the plasma membrane and endoplasmic reticulum, regulates calcium cycling through its binding partner PKA [63]. AKAP-7 has specificity in targeting PKA to ion channels for regulation of both skeletal muscle calcium channels and brain sodium channels [64]. A PKA-binding peptide derived from AKAP-7 blocked voltage-dependent potentiation of calcium channel activity [65]. In IDN, AKAP-7 was upregulated, which may regulate PKA and PKC to promote IDN. Meanwhile, the ROC results (AUC: 0.872; 95% CI 0.805–0.939) illustrated that AKAP-7 could be a good candidate molecular biomarker for IDN.

## Conclusions

In these preliminary discovery experiments, we first implemented the iTRAQ technique to identify plasma gelsolin, collectin-11, PTPRJ, and AKAP-7 as candidate biomarkers in type 2 IDN, which will provide a useful basis for further analysis of the pathogenic mechanism of type 2 diabetic nephropathy.

## Data Availability

All data are stored in the form of an electronic database together and results from analysis in the form of a statistical software report.
